# Genome-wide analysis of *MYB* transcription factor family and *AsMYB1R* subfamily contribution to ROS homeostasis regulation in *Avena sativa* under PEG-induced drought stress

**DOI:** 10.1186/s12870-024-05251-w

**Published:** 2024-07-06

**Authors:** Yang Chen, Aixue Li, Ping Yun, Quan Chen, Dayu Pan, Rui Guo, Han Zhang, Hassan Ahmed Ibraheem Ahmed, Haiying Hu, Yuanying Peng, Cheng Wang, Hongtu Dong, Chaoyang Qiu, Lana Shabala, Sergey Shabala, Bin Luo, Peichen Hou

**Affiliations:** 1 Information Technology Research Center, Beijing Academy of Agriculture and Forestry Sciences, Beijing, 100083 China; 2 Intelligent Equipment Research Center, Beijing Academy of Agriculture and Forestry Sciences, Beijing, 100083 China; 3 School of Biological Sciences, University of Western Australia, Crawley, WA 6009 Australia; 4 International Research Centre for Environmental Membrane Biology, Foshan University, Foshan, 528000 China; 5 Department of Botany, Faculty of Science, Port Said University, Port Said, 42526 Egypt; 6 College of Forestry and Prataculture, Ningxia University, Yinchuan, 750021 China; 7 State Key Laboratory of Crop Gene Exploration and Utilization in Southwest China, Sichuan Agricultural University, Chengdu, 625014 China; 8 College of Life Scienc, Jilin Agricultural University, Changchun, 130118 China

**Keywords:** *Avena sativa*, Drought stress, MYB transcription factors, ROS

## Abstract

**Background:**

The myeloblastosis (*MYB*) transcription factor (TF) family is one of the largest and most important TF families in plants, playing an important role in a life cycle and abiotic stress.

**Results:**

In this study, 268 *Avena sativa MYB* (*AsMYB*) TFs from *Avena sativa* were identified and named according to their order of location on the chromosomes, respectively. Phylogenetic analysis of the AsMYB and *Arabidopsis* MYB proteins were performed to determine their homology, the AsMYB1R proteins were classified into 5 subgroups, and the AsMYB2R proteins were classified into 34 subgroups. The conserved domains and gene structure were highly conserved among the subgroups. Eight differentially expressed *AsMYB* genes were screened in the transcriptome of transcriptional data and validated through RT-qPCR. Three genes in *AsMYB2R* subgroup, which are related to the shortened growth period, stomatal closure, and nutrient and water transport by PEG-induced drought stress, were investigated in more details. The *AsMYB1R* subgroup genes *LHY* and *REV 1*, together with GST, regulate ROS homeostasis to ensure ROS signal transduction and scavenge excess ROS to avoid oxidative damage.

**Conclusion:**

The results of this study confirmed that the *AsMYB* TFs family is involved in the homeostatic regulation of ROS under drought stress. This lays the foundation for further investigating the involvement of the *AsMYB* TFs family in regulating *A. sativa* drought response mechanisms.

**Supplementary Information:**

The online version contains supplementary material available at 10.1186/s12870-024-05251-w.

## Background

Over the last decade, intensified climate change has seriously impacted profitability of agricultural production systems severely impacting crops growth and yields. One of the major constraints is a drought stress that cause crop yields to decrease by 50–70% [1]. Drought stress affects growth and development of plants by reducing water availability. The most significant manifestations of this process are reduced leaf water potential and associated stomatal closure; reduced photosynthesis leading to an imbalance in the source-sink relationship; and increased production of reactive oxygen species (ROS), leading to membrane ester peroxidation and electrolyte leakage. All these processes further disturb plant’s osmotic balance, eventually leading (in severe cases) to plant death [2]. Plants adapt to drought stress by employing a broad range of anatomical (e.g. leaf shape and angle; root system architecture; shoot pubescence and glaucousness) and physiological traits. One of the critical traits in the latter groups is efficient osmotic adjustment that can be achieved by combination of enhanced uptake of inorganic ions (K^+^, Na^+^, Cl^-^) [2] and *de novo* synthesis of organic osmolytes (proline, soluble sugars, alcohols, betaines, etc.) [3]. Plants also synthesize ABA to close stomata and reduce water loss [4] as well as control water loss by adjusting stomatal density [5]. In addition, plants regulate ROS homeostasis by synthesizing antioxidant enzymes and antioxidants to scavenge ROS [6]. All above processes are controlled at both transcriptional and post-translational levels and rely heavily on numerous transcriptional factors (TFs). Among major classes of TFs are MYB, APETALA2/ethylene-responsive element binding protein (AP2/EREBP), basic helix-loop-helix (bHLH), basic region/leucine zipper motif (bZIP), NAM, ATAF1, ATAF2 and CUC2 (NAC), WRKYGQK sequence (WRKY) and zinc finger protein (ZFP) [7].

*Avena sativa* (*A. sativa*) is an allohexaploid crop (AACCDD, 2n = 6x = 42) of the family *Poaceae* in the genus *Avena*, which ranks sixth in world cereal production [8]. *A. sativa* is an important grain and forage grass, functionally classified as a food rich in soluble fiber, β-glucan, lipid, protein, and antioxidants [8–10] *A. sativa* also possesses good adaptability to various soil environments [10, 11]. Although *A. sativa* has relatively high abiotic stress tolerance, it is usually grown in areas where other crops cannot cope with climate extremes [12], and therefore, its yield is affected by environmental stress. Therefore, more strategies are needed to improve the stress resistance of *A. sativa*, especially drought resistance, because most areas where *A. sativa* is grown are prone to severe drought stress [12, 13]. Therefore, studying the molecular mechanisms of drought tolerance in *A. sativa* is particularly important for breeding programs and coping with future climate environments.

Under drought stress, oats synthesize ABA to reduce stomatal conductance and close stomata to reduce water loss [14]. This comes with some penalties such as reduced photosynthetic efficiency, reduce nutrient consumption, reduction in growth rate, shortened life cycle and accelerated aging [13]. Oats also synthesize organic compounds such as proline and glycine betaine (GB) to osmotically adjust to drought conditions [15]; this process, however, comes with high carbon cost and also on expense of growth [16]. However, little is known about the regulation of these pathways by TFs.

The recently reported high-quality genome and annotation data of *A. sativa* allohexaploid provide important references for the study of TFs [9, 17]. Of a specific interest are *MYB* TFs, as *MYB* genes play important roles in cell cycle, metabolism, abiotic stress response [18–22]. MYB TFs possess highly conserved MYB DNA binding domain at N-terminal, which typically consists of one to four imperfect tandem repeats (R). The repeats consist of 51–53 amino acid residues that form a helix-turn-helix (HTH) structure that interacts with target DNA [23]. In addition, this conserved structure of repeats comprises regularly interval triplet tryptophan residues, which aggregate to form a hydrophobic core. The repeats of MYB DNA binding domain are named as R1, R2, and R3 based on the similarity to Myb-c protein [24]. The *MYB* genes can be classified into four categories according to the number of MYB repeat and the characteristic of MYB sequences, including MYB-related (MYB1R), R2R3-MYB (MYB2R), R1R2R3-MYB (MYB3R), and atypical MYB (MYB4R) [25]. The *MYB* genes have been systematically studied in *Arabidopsis thaliana* [25], *Diospyros oleifera* [26], and *Melastoma candidum* [27].

As commented above, drought tolerance traits are closely associated with plant’s ability to maintain redox balance and prevent oxidative stress damage. Drought stress leads to overaccumulation of various types of ROS in plant cells, such as superoxide (O_2_^-^), hydrogen peroxide (H_2_O_2_), singlet oxygen (^1^O_2_), and hydroxyl radicals (OH∙) [28], causing damage to cellular components (proteins, nucleic acids, and lipids) and triggering programmed cell death. Plant scavenge ROS through the synergistic action of enzymatic and non-enzymatic antioxidant mechanisms. The enzymatic antioxidants include superoxide dismutase (SOD), catalase (CAT), glutathione reductase (GR), and ascorbate peroxidase (APX). Non-enzymatic antioxidants include β-carotene, α-tocopherol, ascorbic acid, glutathione, anthocyanins, and flavonoids [2, 29, 30]. Importantly, ROS plays a double-edged sword role, acting as a signaling molecule in mild abiotic stress signal transduction [31, 32] and causing oxidative stress to key macromolecules under more severe conditions.

It was shown that expression of *CIRCADIAN CLOCK ASSOCIATED* (*CCA1*) gene was closely related to redox regulation, and mutations in the core clock regulatory factor CCA1 have been shown to affect ROS homeostasis and the transcriptionally regulated expression of tolerance to oxidative stress of ROS responsive ROS-responsive genes [18]. It was suggested that CCA1 is the main regulator of ROS homeostasis, and ROS functions as an input signal that affects the transcriptional output of the clock. CCA1 belongs to *REV* family, alongside with some other proteins related to the biological clock such as *LATE ELONGATED HYPOCOTYL* (*LHY*), and 9 *REVEILLE* (*RVE*) genes. This family consists of two subgroups: the first subgroup includes *CCA1*, *LHY*, *RVE 1*, *2*, *7*, and *RVE 7-like*, and the second subgroup includes *RVE 3*, *4*, *5*, *6*, and *8* [33], with all proteins having a single highly conserved MYB/SANT domain.

Herein, 268 members of *AsMYB* TFs were identified based on the genome and annotation data of *A. sativa.* Further, comprehensive bioinformatic analysis was performed in terms of chromosome location, phylogenetic analysis, conserved domain, gene structure, and gene duplication of the *AsMYB* genes. The expression levels of 8 *AsMYB* genes in *A. sativa* roots under PEG-induced drought stress at different time points were analyzed based on transcriptome data and RT-qPCR, with consistent results between two data sets. Three genes in *AsMYB2R* subgroup, which are related to the shortened growth period, stomatal closure, and nutrient and water transport by PEG-induced drought stress, were investigated in more details. These findings demonstrate that circadian clock key genes *LHY* and *RVE1* of the *AsMYB1R* subfamily, along with non-enzymatic antioxidant *GST* genes, jointly regulate ROS homeostasis in *A. sativa* plants.

## Results

### Identification of MYB TFs and physicochemical properties in *A. sativa*

**Fig. 1:**
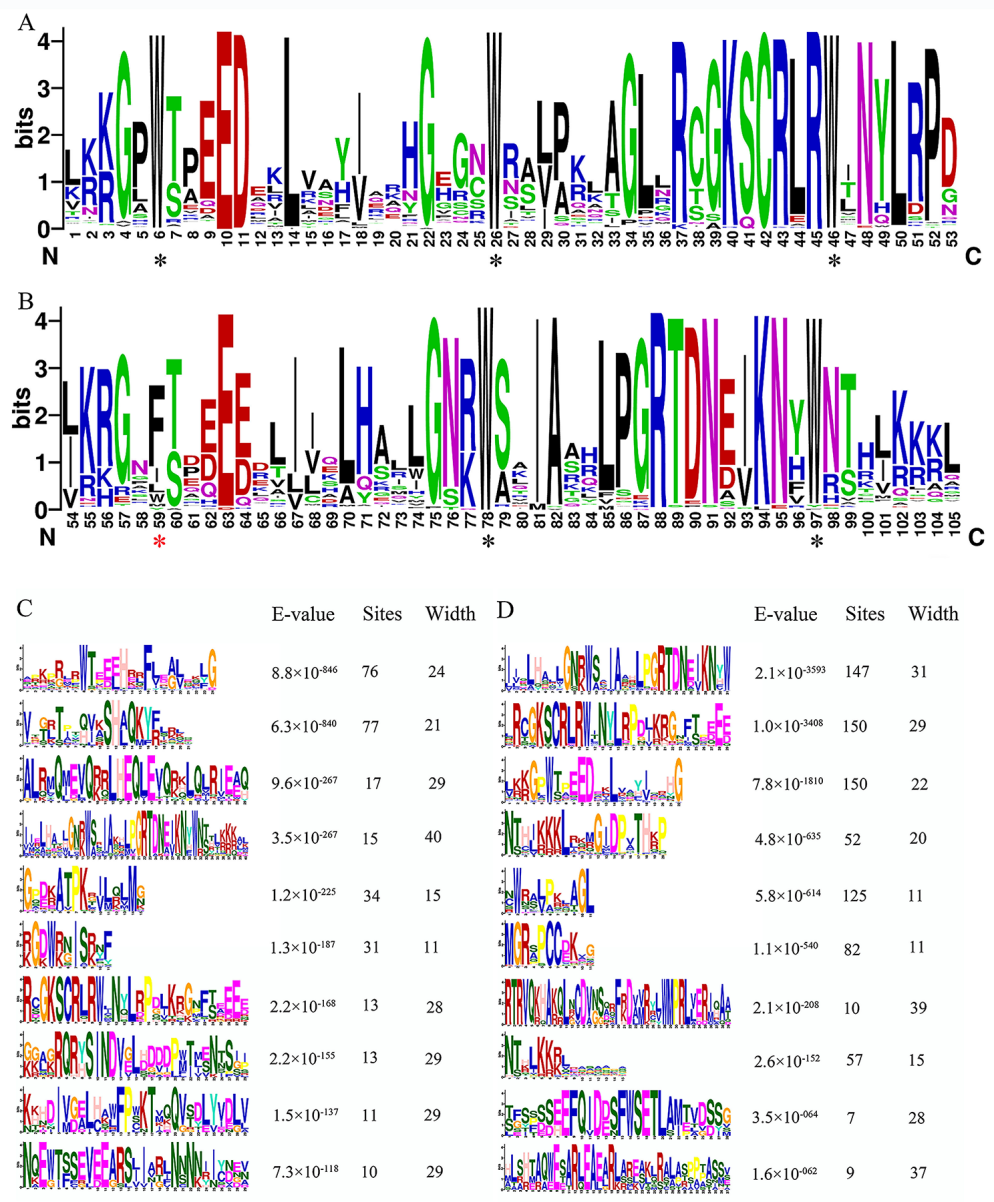
The conserved amino acid sequences logo of AsMYB2R proteins. (**A**) The sequence logo of the R2 conserved domain includes three conserved tryptophan residues. (**B**) The sequence logo of the R3 conserved domain. The typically conserved tryptophan amino acid sites were labeled by black *, and red * represents the substituted amino acid site in the R3 domain. (**C**) The detail information of AsMYB1R TFs conserved motifs includes E-value, site and width. (**D**) The detail information of AsMYB2R TFs conserved motifs includes E-value, site and width

*AsMYB* genes were initially screened by aligning the HMM and alignment file of MYB (PF00249) against the protein data using HMMER software. Subsequently, CD-HIT software, NCBI-CDD, and SMART database were successively used to obtain 268 *AsMYB* TFs which removed redundant sequences and possessed complete MYB structure. Based on the number and characteristics of MYB DNA binding repeats, the *AsMYB* genes were classified into three categories: 112 *AsMYB1R* TFs, 151 *AsMYB2R* TFs, and 5 *AsMYB3R* TFs. Since R1, R2, and R3 are different in amino acid structure and R2 and R3 are highly conservative, the conserved region of AsMYB2R proteins was aligned and visualized, as shown in Fig. 1. The characteristic sequence of R2 domain was [-W-(X19)-W-(X19)-W-], and R3 domain was [-F/I/L/W-(X18)-W-(X18)-W-]. Highly conservative structure of R2 and R3 were consistent with previous reports in *A. thaliana*, which proved the accuracy of *AsMYB2R* genes. It was notability that the first tryptophan (W) residue of the R3 repeat was replaced by Leucine (L), Isoleucine (I), or Phenylalanine (F) residues, which was a common phenomenon in plant R2R3-MYBs [34]. Hydrophobic residues of L, I, and F have proved that they can substitute for W and maintain the function of the MYB domain, at least in terms of DNA binding, which has been proved in animals [35]. The multiple sequence alignment plot of AsMYB2R proteins showed that they are highly conserved (Fig. 1) and similar to other species such as *Hibiscus cannabinus* [36], *Linum usitatissimum* [37], and *Pyrus bretschneideri* [38], indicating the conserved nature of *MYB* genes during the evolution of the plant lineage. The physicochemical properties of AsMYB proteins are shown in Table S2. The size of AsMYB proteins ranges from 126 to 1942 amino acids, with a predicted molecular weight ranging from 13.87 to 208.67 KDa. Since only 6 AsMYB proteins have a protein size of more than 1000 amino acids, the average molecular weight was 40.33 KDa. The predicted isoelectric point (pI) ranges from 4.51 to 11.69, with an average pI of 7.06. Surprisingly, only circa 41.4% of AsMYB proteins translated basic proteins. Only 9 AsMYB proteins were predicted to belong to stable proteins (instability index less than 40), indicating that the AsMYB proteins as a whole was in an unstable state and the proteins were easily degraded. The grand average of hydropathicity of AsMYB proteins was less than 0 (-1.155 to -0.154) except AsMYB1R035 protein (0.065), which indicated that almost all of AsMYB proteins were hydrophilic. By using the WOLF PSORT online tool, subcellular localization analysis showed that the majority of the *AsMYB* genes were predicted in the nucleus, with a small set of them were predicted to localize in other subcellular locations, such as chloroplast, cytoplasm, and mitochondrion (Table S2).

### Chromosome localization and phylogenetic tree construction in *A. sativa*

**Fig. 2:**
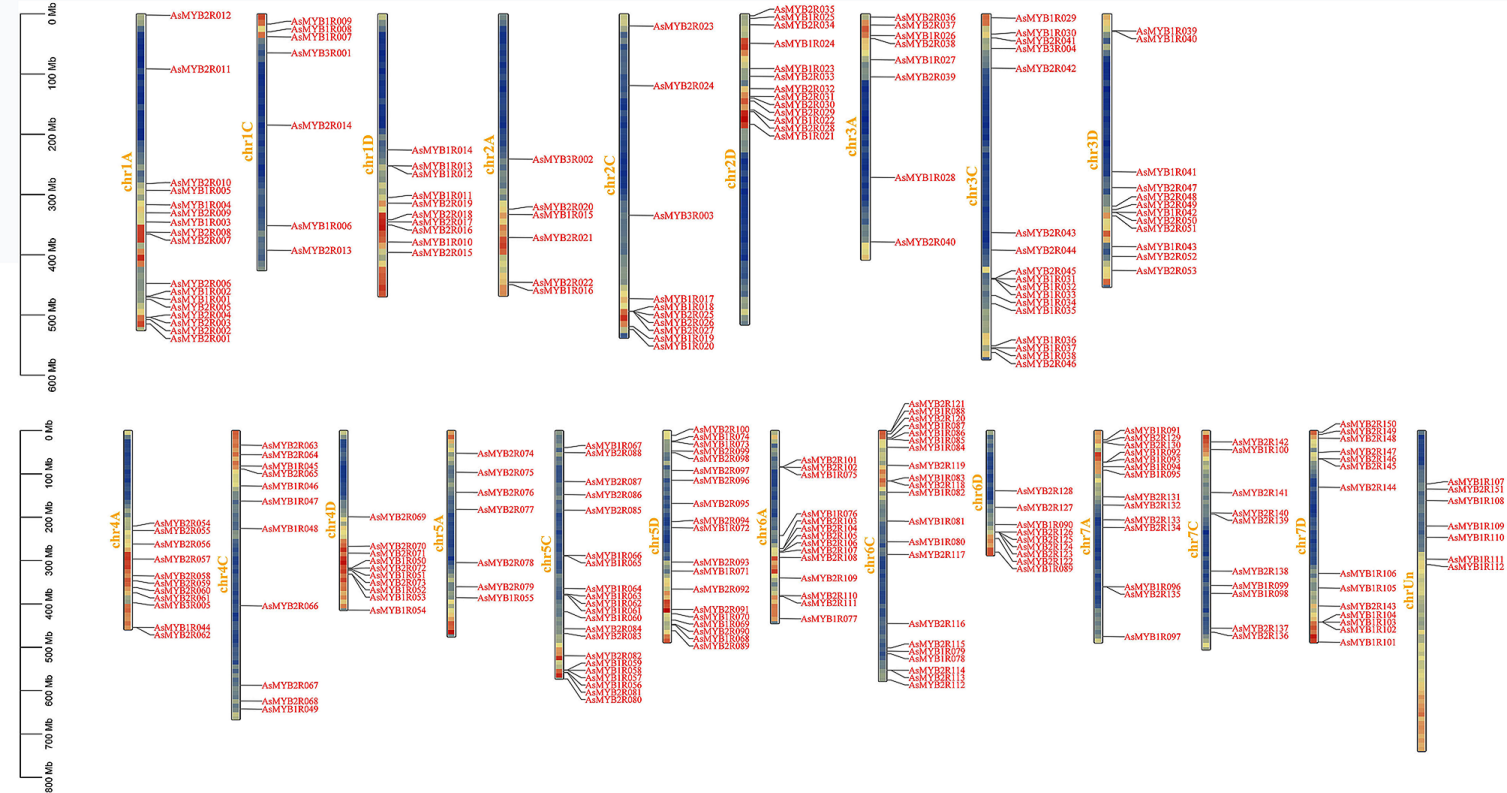
Distribution of *AsMYB* TFs on *A. sativa* chromosomes. The chromosomal position of each *AsMYB* TFs was mapped to the *A. sativa* genome. ChrUn represents genes that have failed to assemble onto the chromosomes

**Fig. 3:**
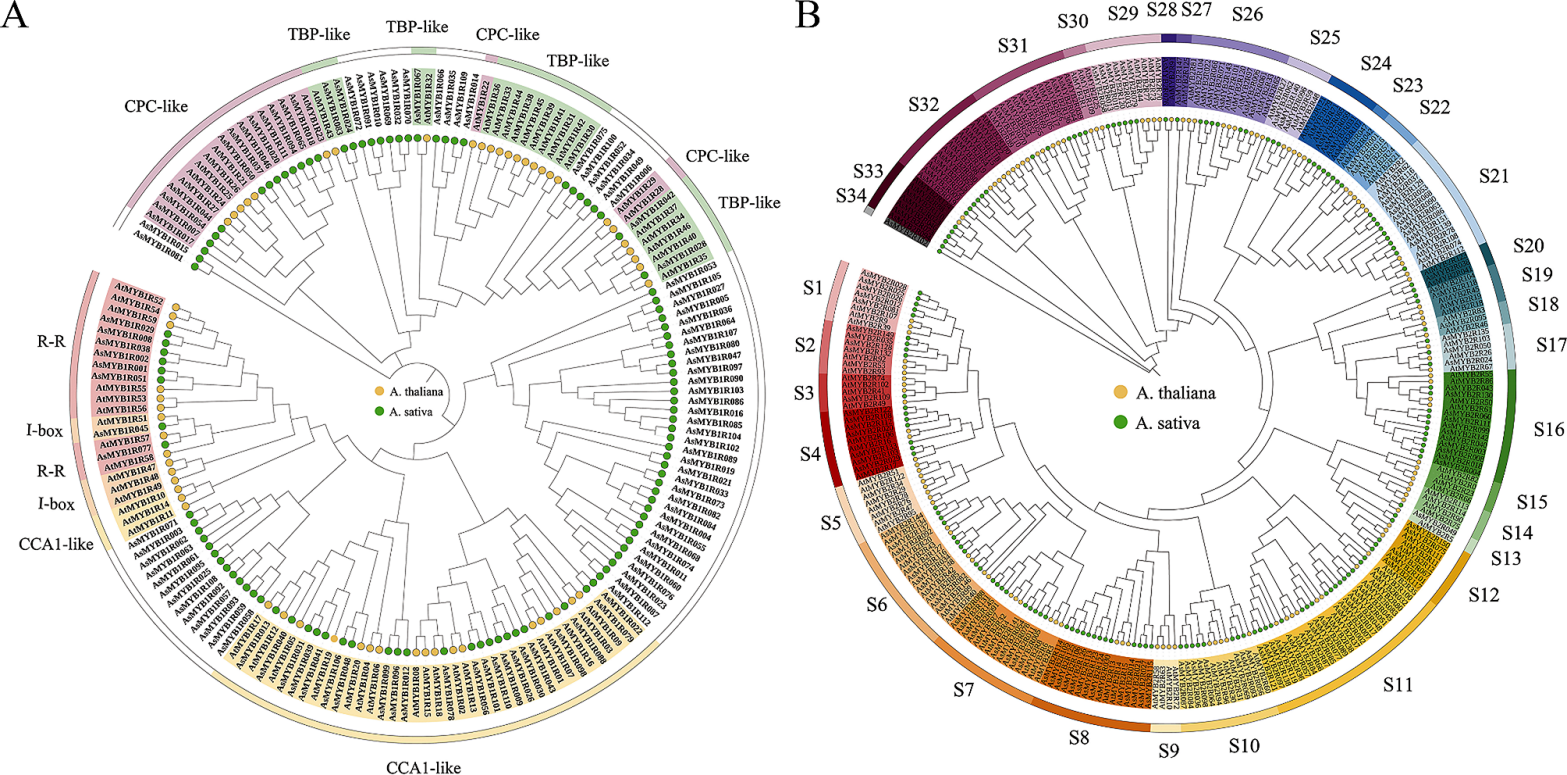
The phylogenetic tree plots of *AtMYB* and *AsMYB* TFs. (**A**) The phylogenetic tree plot of *AsMYB1R* and *AtMYB1R* TFs. The *AsMYB1R* TFs are divided into 5 subgroups (CCK1-like, CPC-like, TBP-like, I-box-binding-like, and R-R). (**B**) The phylogenetic tree plot of *AsMYB2R* and *AtMYB2R* TFs. The *AsMYB2R* TFs are divided into 34 subgroups (S1-S34). Yellow and green dots represent *AtMYB* TFs and *AsMYB* TFs, respectively

*AsMYB* genes localization plot was mapped according to the annotation data of *A. sativa*. *AsMYB* genes of three categories were named according to their order of location on the chromosomes, respectively. Genes that were not assembled onto the chromosome were defined as ChrUn [39]. In *AsMYB* genes localization plot (Fig. 2), *AsMYB1R* TFs were named as *AsMYB1R001*-*AsMYB1R112*, with six genes located on ChrUn. *AsMYB2R* TFs were named as *AsMYB2R001*-*AsMYB2R151*, and *AsMYB3R* TFs were named as *AsMYB3R001*-*AsMYB3R005*. Particular chromosomes were found; of these, Chr1C had the lowest gene density and Chr4D had the highest gene density, *AsMYB* TFs were unevenly distributed in each chromosome [40]. The detailed information on the number of *AsMYB* genes in different chromosomes is shown in Table S3. The result indicated that the *AsMYB* TFs underwent duplication events (Fig. S1). Since 98.13% *AsMYB* TFs were made up of *AsMYB1R* TFs and *AsMYB2R* TFs, the Neighbor-Joining phylogenetic trees were constructed using MEGA software, respectively. As shown in Figs. 3A and 112 AsMYB1R proteins and 59 AtMYB1R proteins were aligned, and phylogenetic tree of MYB-related revealed 5 subgroups (CCK1-like, CPC-like, TBP-like, I-box-binding-like, and R-R), of which TBP-like and I-box-binding-like in *A. sativa* contracted more than double. Similar to *A. thaliana*, the CCK1-like subgroup has the most members of 22 *AsMYB1R* TFs, and the I-box-binding-like subgroup has the least members of 1 *AsMYB1R* TFs. The phylogenetic tree of AsMYB2R proteins was shown in Fig. 3B, which contained 151 AsMYB2R proteins and 126 AtMYB2R proteins. In Figs. 3B and 34 subgroups were divided and the amount of *AsMYB2R* genes were varied in each subgroup (the number from 1 to 17). The distribution of *A. sativa* and *A. thaliana* for the same subgroups was also greatly different. Five subgroups (S4, S8, S11, S16, and S33) of *A. sativa* expanded more than two-fold, while S3 of *A. sativa* contracted more than twice. In addition, the subgroups included the member of *A. thaliana* (S5, S9, S14, S15, and S30) and the member of *A. sativa* (S23 and S34). This implied ancestral gene duplication and loss events [41]. More information is available from Table S4.

### Conserved motif, gene structure, and cis-acting in *A. sativa*

**Fig. 4:**
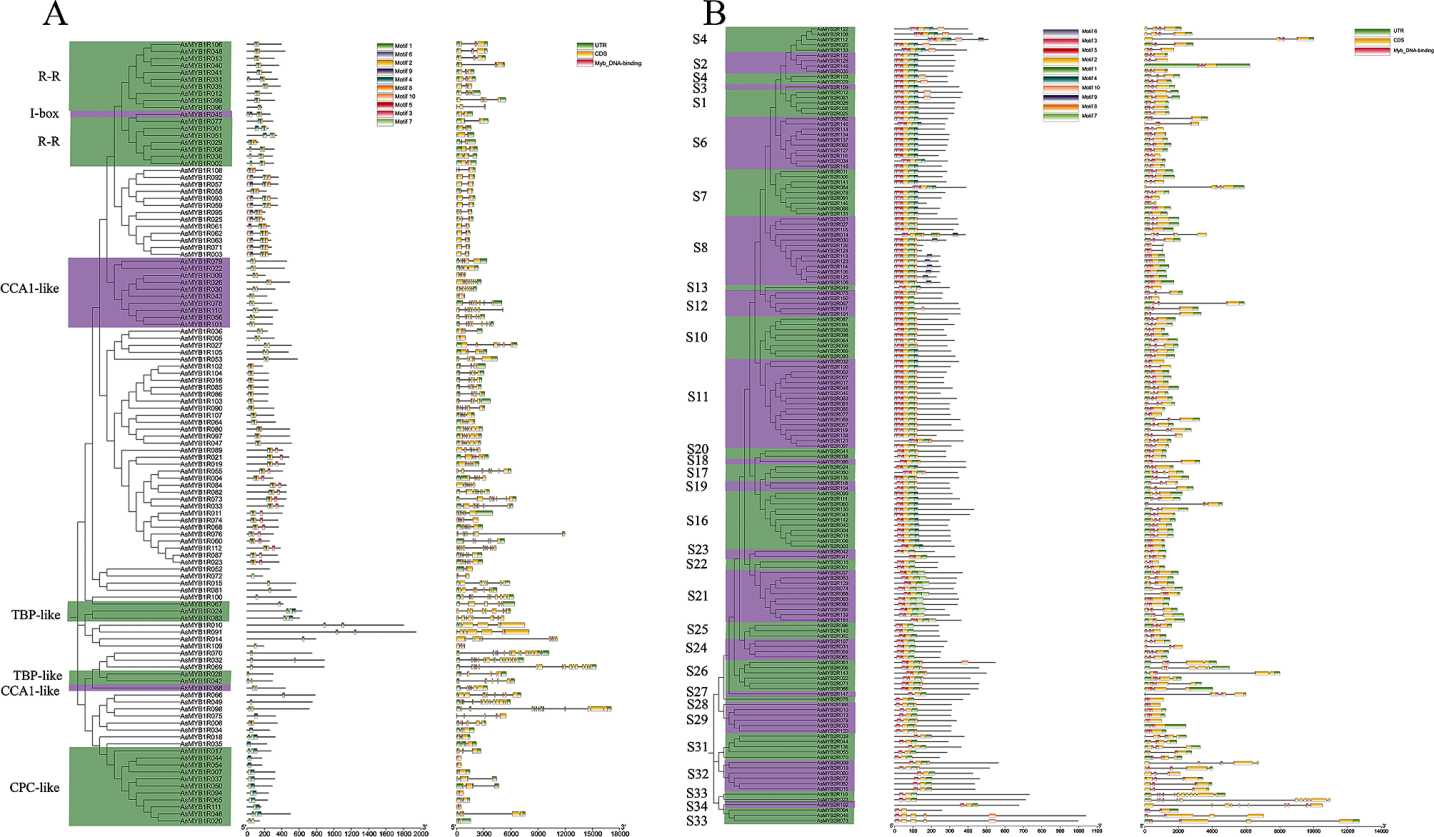
Conserved motif, gene structure, and phylogenetic tree plots of *AsMYB* TFs. (**A**) The plot of *AsMYB1R* TFs; (**B**) The plot of *AsMYB2R* TFs. Conserved motifs are highlighted with different colored backgrounds and numbers, and their position in each MYB sequence was determined. The CDS, UTR and Myb DNA-binding site were mapped on the genes and indicated by different colors, respectively

To further understand the diversity of *AsMYB1R* and *AsMYB2R* TFs, the conserved motifs were analyzed by using the MEME online program, and ten conserved motifs were investigated. The motif and structure plots of *AsMYB* genes are shown in Fig. 4. For *AsMYB1R* genes (Fig. 4A), the number of conserved motifs varied greatly among subgroups (from 1 to 6), the subgroups (I-box-binding-like and R-R) constituted motifs 1, 2, and 6 in common. CCK1-like subgroup constituted of motifs 1 and 2, which indicate that the members in the same subgroups have a similar function. The motif and structure plot of *AsMYB2R* genes (Fig. 4B) showed more motifs than *AsMYB1R* genes, and all numbers of *AsMYB2R* genes have more than three motifs besides *AsMYB2R094*. The gene structure analysis showed some incomplete UTRs or too-long regions of some genes, which may be caused by genome assembly. The cis-acting elements of *AsMYB* genes were analysed (Fig S2), 24 cis-elements mainly related to auxin response, abscisic acid response, gibberellin response, and light response were identified.

### Gene duplication and collinearity information in *A. sativa*

**Fig. 5:**
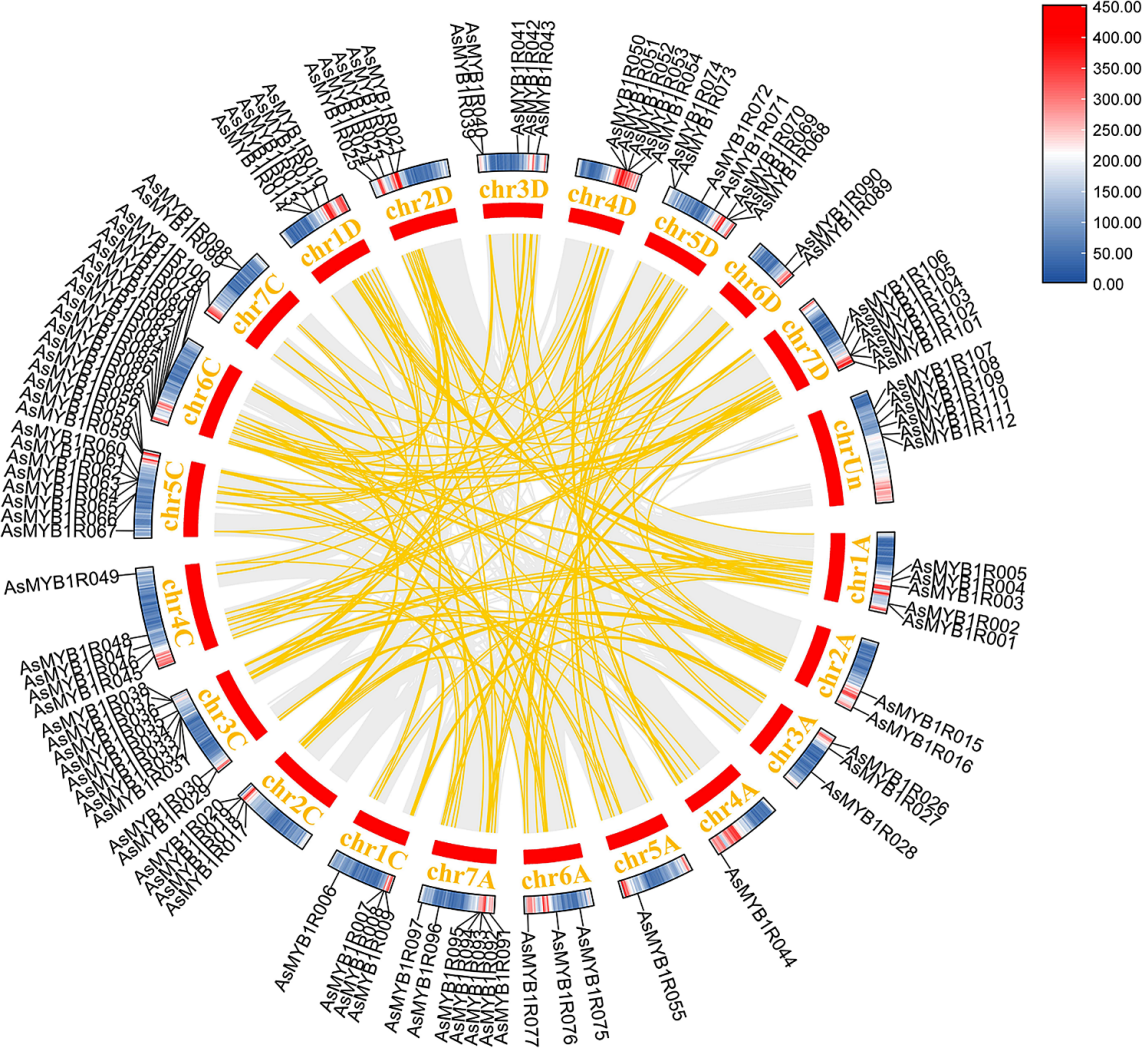
Gene duplication plots of *AsMYB1R* TFs. The gray line represents all duplication events in *A. sativa* genome, the yellow line represents the *AsMYB1R* TFs duplication events, and the heat map represents the gene density in *A. sativa* genome

**Fig. 6:**
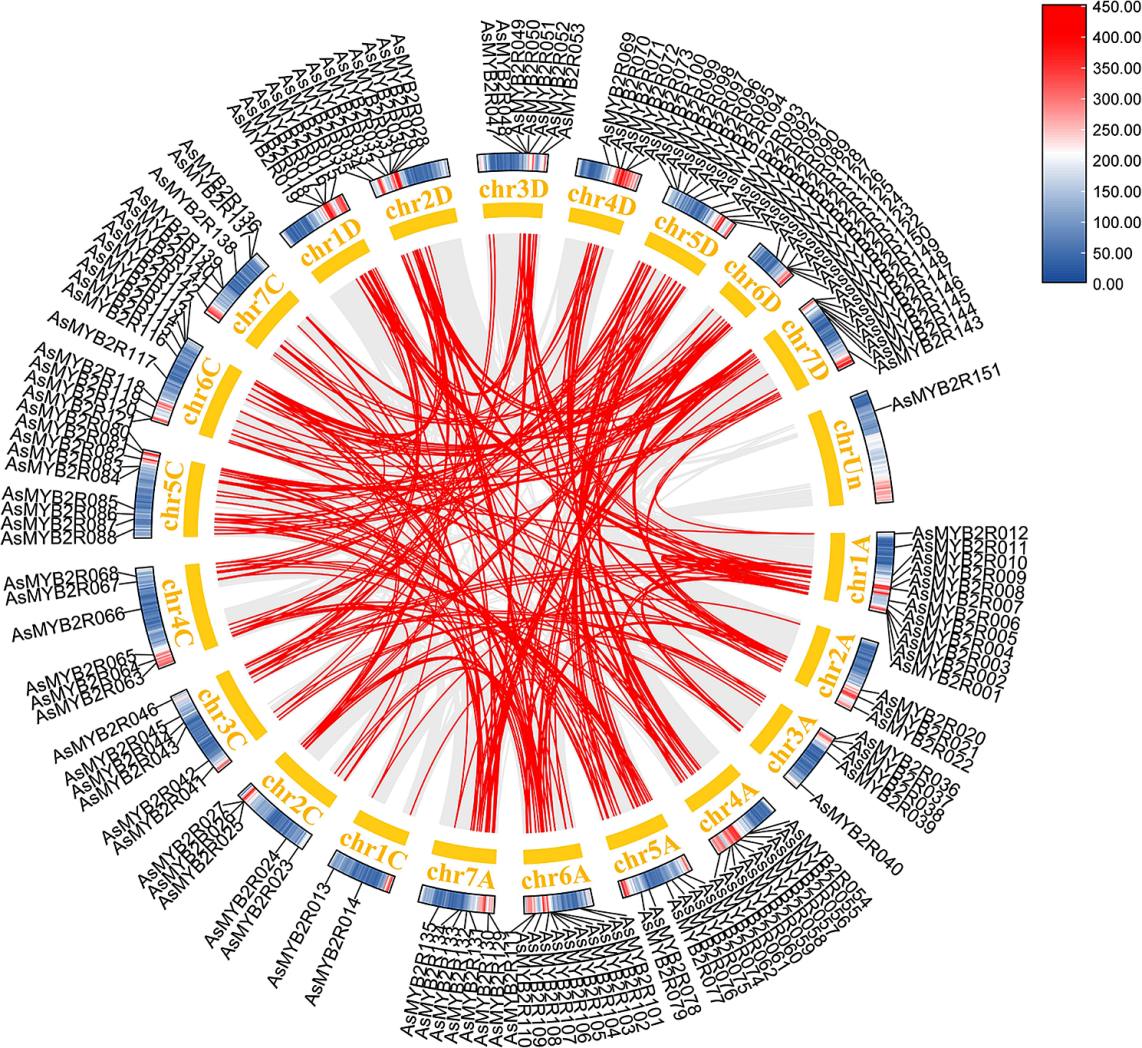
Gene duplication plots of *AsMYB2R* TFs. The gray line represents all duplication events in *A. sativa* genome, the red line represents the *AsMYB2R* TFs duplication events, and the heat map represents the gene density in *A. sativa* genome

**Fig. 7:**
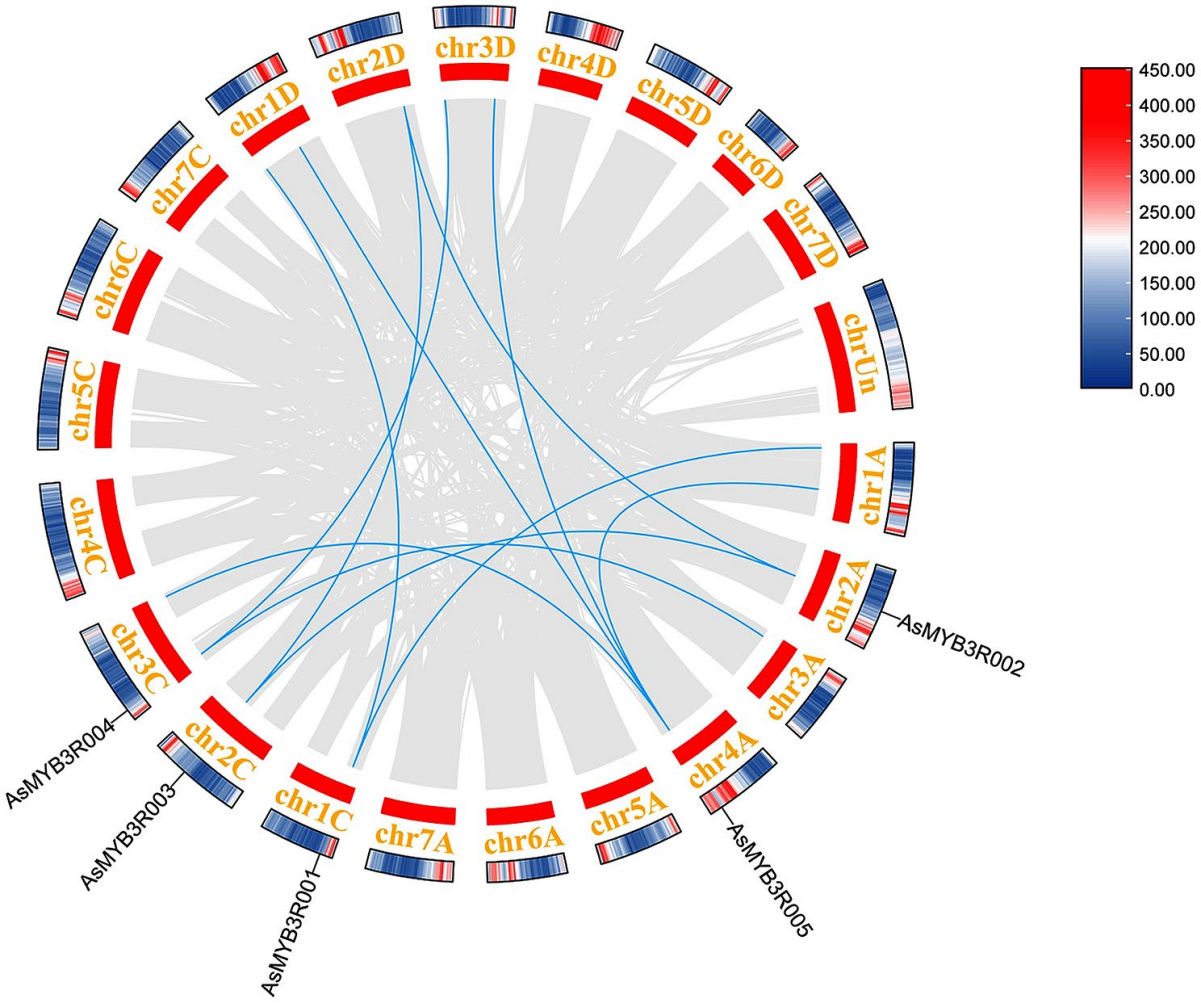
Gene duplication plots of *AsMYB3R* TFs. The gray line represents all duplication events in *A. sativa* genome, the blue line represents the *AsMYB3R* TFs duplication events, and the heat map represents the gene density in *A. sativa* genome

Gene duplication is a common phenomenon in the process of plant evolution that played an important role in the gene expansion [42]. Here, the duplication event plots of the *AsMYB1R* TFs, *AsMYB2R* TFs, and *AsMYB3R* TFs were identified by MCScanx methods to determine the gene duplication events, respectively. The gene segmental duplication event could serve as an important access for plants to acquire new genes and contribute to the gene family expansion as well. As shown in Fig. 5, a total of 91 *AsMYB1R* genes were identified to form 192 segmental duplication pairs. The ends of 61 gene pairs were comprised by *AsMYB1R* genes, where 12 gene pairs were connected by *AsMYB1R* and *AsMYB2R* genes, residual gene pairs were one end being *AsMYB1R* genes, and the other joined the gene of non-*AsMYB* genes. The gene duplication events of 129 *AsMYB2R* genes are shown in Fig. 6. In addition, 308 gene pairs were discovered, where 167 gene pairs consisted of *AsMYB2R* genes, the ends of the 12 gene pairs are *AsMYB1R* and *AsMYB2R* genes, and the ends of 2 gene pairs are *AsMYB2R* and *AsMYB3R* genes. Furthermore, Fig. 7 shows 11 segmental duplication pairs formed by 5 *AsMYB3R* genes belonging to *AsMYB3R* TFs, including 1 gene pair for *AsMYB3R* genes, and 2 gene pairs comprised of *AsMYB2R* and *AsMYB3R* genes. At the same time, the Ka/Ks values of all gene pairs were less than 1, indicating that *AsMYB* genes in *A. sativa* underwent purify selection during evolution [43]. More information is given in Table S5.

### Expression of *AsMYB* TFs under drought stress in *A. sativa*

**Fig. 8:**
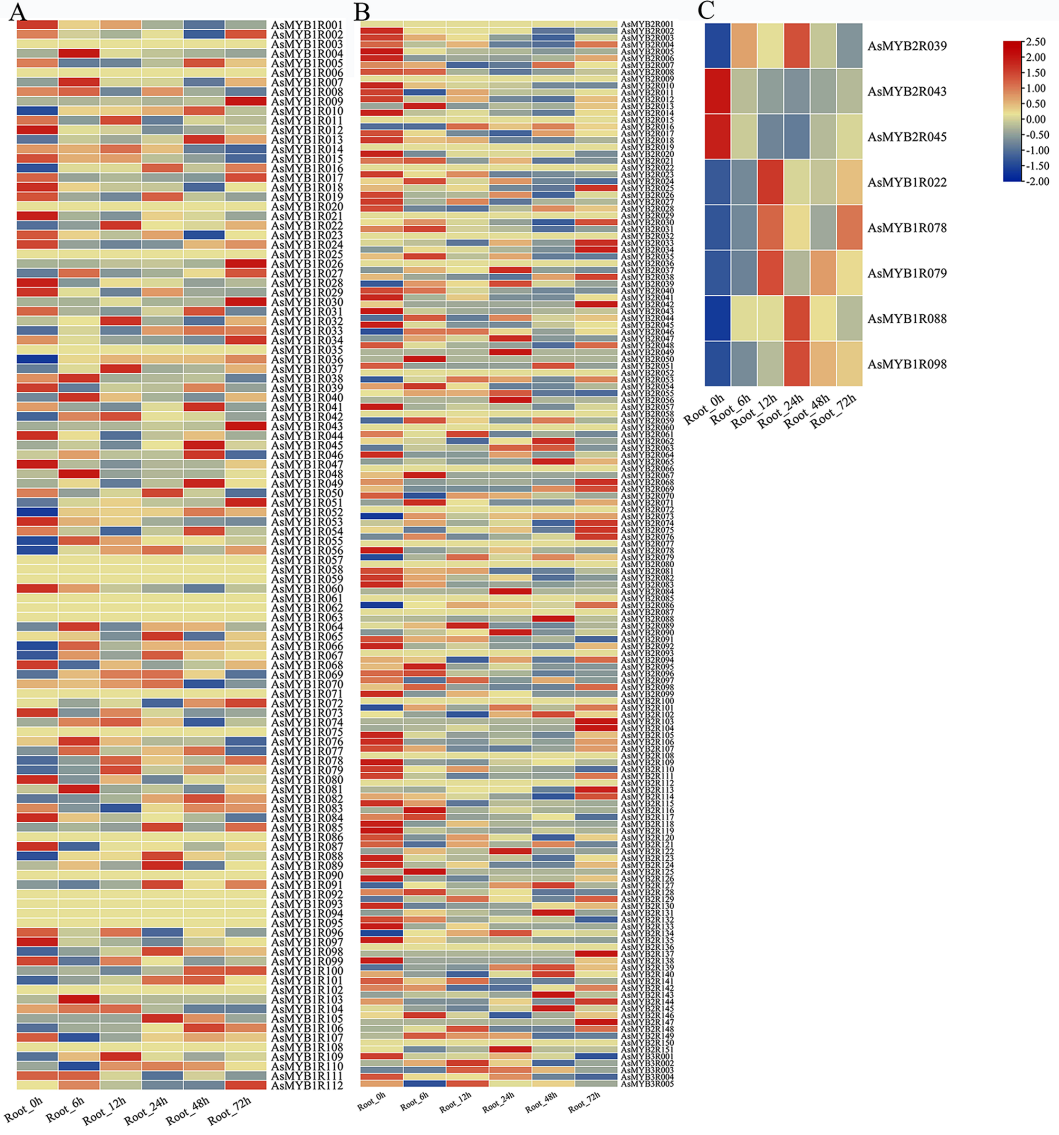
Transcriptional abundance plots of *AsMYB* TFs. (**A**) The heatmap showed the expression profile of *AsMYB1R* TFs; (**B**) The heatmap showed the expression profile of *AsMYB2R* and *AsMYB3R* TFs; (**C**) The heatmap showed the expression profile of 8 differential expression genes. Rows represent the different time samples (0–72 h), the columns represent the different *AsMYB* genes

There is always an association between gene expression patterns and function, and how *AsMYB* genes can regulate gene expression when plants suffer from environmental changes. To explore *AsMYB* genes related to drought response, transcriptional abundance of *AsMYB* genes under drought stress was studied using transcriptome data (https://www.ncbi.nlm.nih.gov/bioproject/PRJNA1056521/). During the growth and development of roots under drought conditions, the data of 44 *AsMYB* genes was not detected in all treatment groups, and there was no significant difference in the expression pattern of the other 216 *AsMYB* genes. Finally, eight differentially expressed *AsMYB* genes (DEGs) were detected. This included five *AsMYB1R* TFs (*AsMYB1R022*, *AsMYB1R078*, *AsMYB1R079*, *AsMYB1R088*, and *AsMYB1R098*) and three *AsMYB2R* TFs (*AsMYB2R039*, *AsMYB2R043*, and *AsMYB2R045*). Figure 8 shows the expression levels of 268 *AsMYB* genes transcriptome data. The expression patterns of different *AsMYB1R* genes, *AsMYB2R* and *AsMYB3R* genes show significant differences at all time stages (Fig. 8A and B). The 8 DEGs of *AsMYB* genes were extracted to display detailed transcriptome abundance information. As shown in Fig. 8C, *AsMYB2R* and *AsMYB1R* genes exhibit differential gene expression patterns at different time points of PEG treatment. For *AsMYB2R* genes, the *AsMYB2R039* was only upregulated in the middle stage (24 h) of drought stress treatment, while AsMYB2R043 and AsMYB2R045 show a downward trend at all five time points after drought treatment. In *AsMYB1R* genes, *AsMYB1R022*, *AsMYB1R078*, and *AsMYB1R079* were upregulated in the early stage (12 h) of drought treatment, followed by a decrease in the expression levels of the three genes. However, *AsMYB1R088* and *AsMYB1R098* were significantly upregulated in the middle stage (24 h) of drought treatment, followed by a decrease in expression levels in the later stages (48 and 72 h) of drought treatment.

**Fig. 9:**
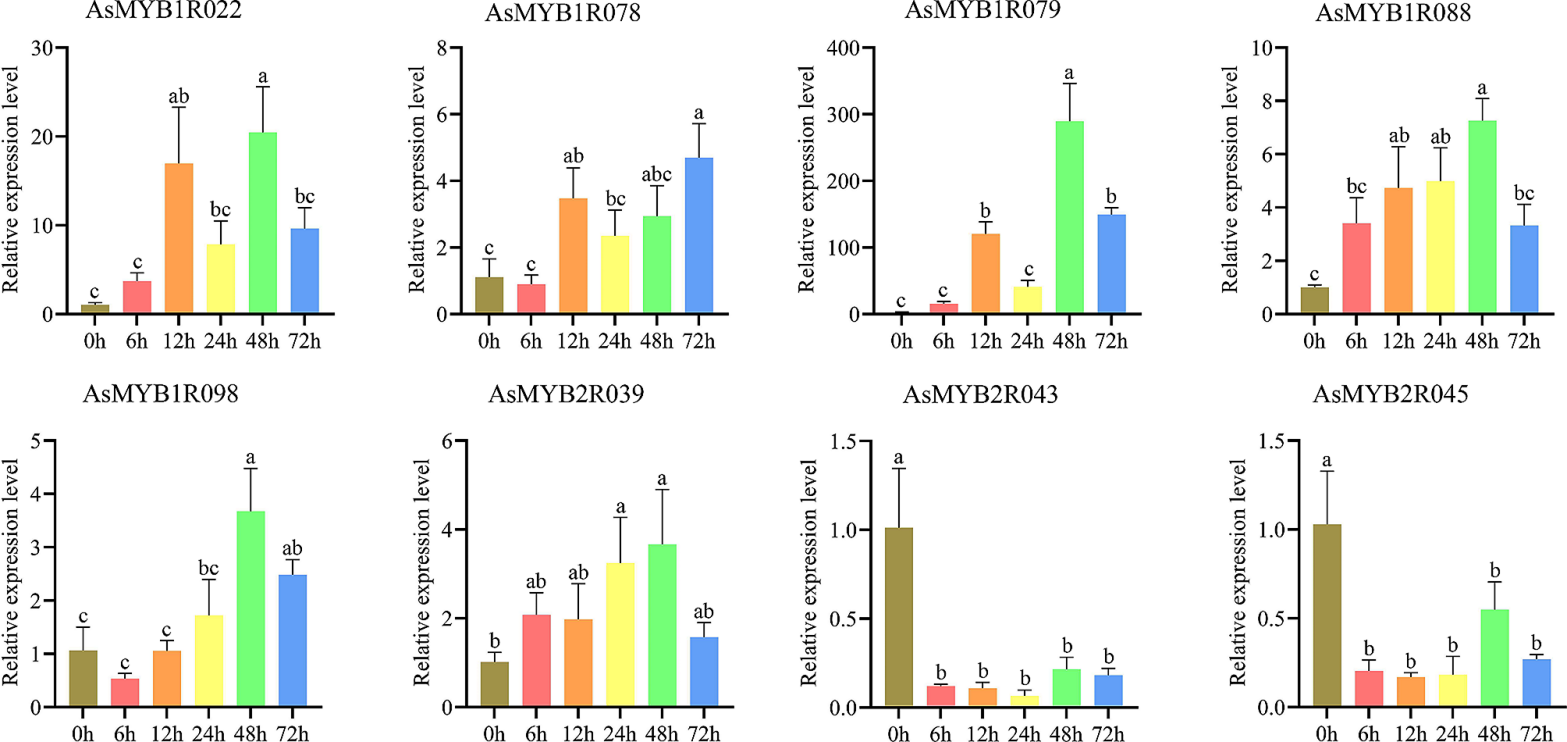
The RT-qPCR plots of 8 differentially expressed genes. Relative expression level plots of 8 *AsMYB* genes following PEG treatment as determined by RT-qPCR. The Y-axis and X-axis indicated relative expression level and six time points of PEG treatment, respectively. The relative expression level of genes at 0 h was taken as 1, and were calculated by normalization method. Mean ± SD (Standard Deviation) was obtained from three biological and three technical replicates. The error bars indicate standard deviation. Different letters indicate significant differences, and the same letters represent no significant differences at the 0.05 level

We then used RT-qPCR approach to validate this data. It was found that *AsMYB2R* genes, *AsMYB2R043* and *AsMYB2R045* had lower transcriptional levels at 5 time points after PEG treatment compared to 0 h (Fig. 9), which is consistent with the transcriptome data (Fig. 8C). The gene expression trend of *AsMYB2R039* gene at 5 time points after PEG treatment was also similar to that from the transcriptome (Fig. 8C). For *AsMYB1R* genes, the gene expression levels of *AsMYB1R022* were upregulated at 5 time points after PEG treatment (Fig. 9), which was consistent with the trend in the transcriptome (Fig. 8C). However, the gene expression levels of *AsMYB1R078*, *AsMYB1R079*, *AsMYB1R088*, and *AsMYB1R098* were basically consistent with the trend in the transcriptome (Fig. 8C). Hence, despite the RT-qPCR validation of *AsMYB1R* and *AsMYB2R* gene expressions were not completely consistent with the multiples in the transcriptome (Fig. 8C), the overall trends were similar.

### Stomatal aperture, H_2_O_2_ and GST quantification

**Fig. 10:**
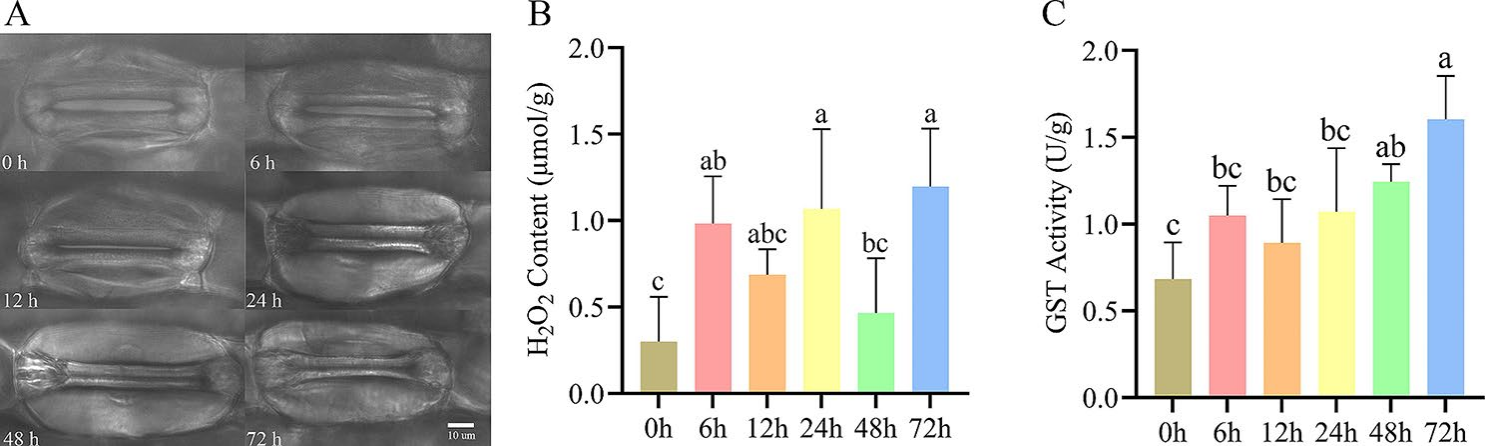
Stomatal aperture, H_2_O_2_ content and GST activity plots. (**A**) The stomatal aperture state at 6 time points (0–72 h) of *A. sativa*; The size of scale bar is 10 μm. (**B**) The analytic result of H_2_O_2_ content. Mean ± SD (Standard Deviation) was obtained from six biological replicates. The error bars indicate standard deviation. Different letters indicate significant differences, and the same letters represent no significant differences at the 0.05 level; (**C**) The analytic result of GST activity. Mean ± SD (Standard Deviation) was obtained from six biological replicates. The error bars indicate standard deviation. Different letters indicate significant differences, and the same letters represent no significant differences at the 0.05 level

After 6 h of PEG treatment, it was observed that the guard cells of *A. sativa* began to contract, and the stomata began to closure. They continued to closure at the following 12, 24, and 48 h. However, after 72 h, the stomata were almost closed (Fig. 10A). The stomata size data also showed a decrease (Fig S3B).

The H_2_O_2_ content in the roots of *A. sativa* showed some fluctuation at 5 time points after PEG treatment, mimicking diurnal patters, with the overall trend for increase, with 5-fold higher H_2_O_2_ content at 72 h compared to 0 h (Fig. 10B). The GST activity in the roots also increased significantly (Fig. 10C), with highest value recorded for 72 h timepoint.

### Changes in the expression levels of *GST* family genes by PEG treatment

**Fig. 11:**
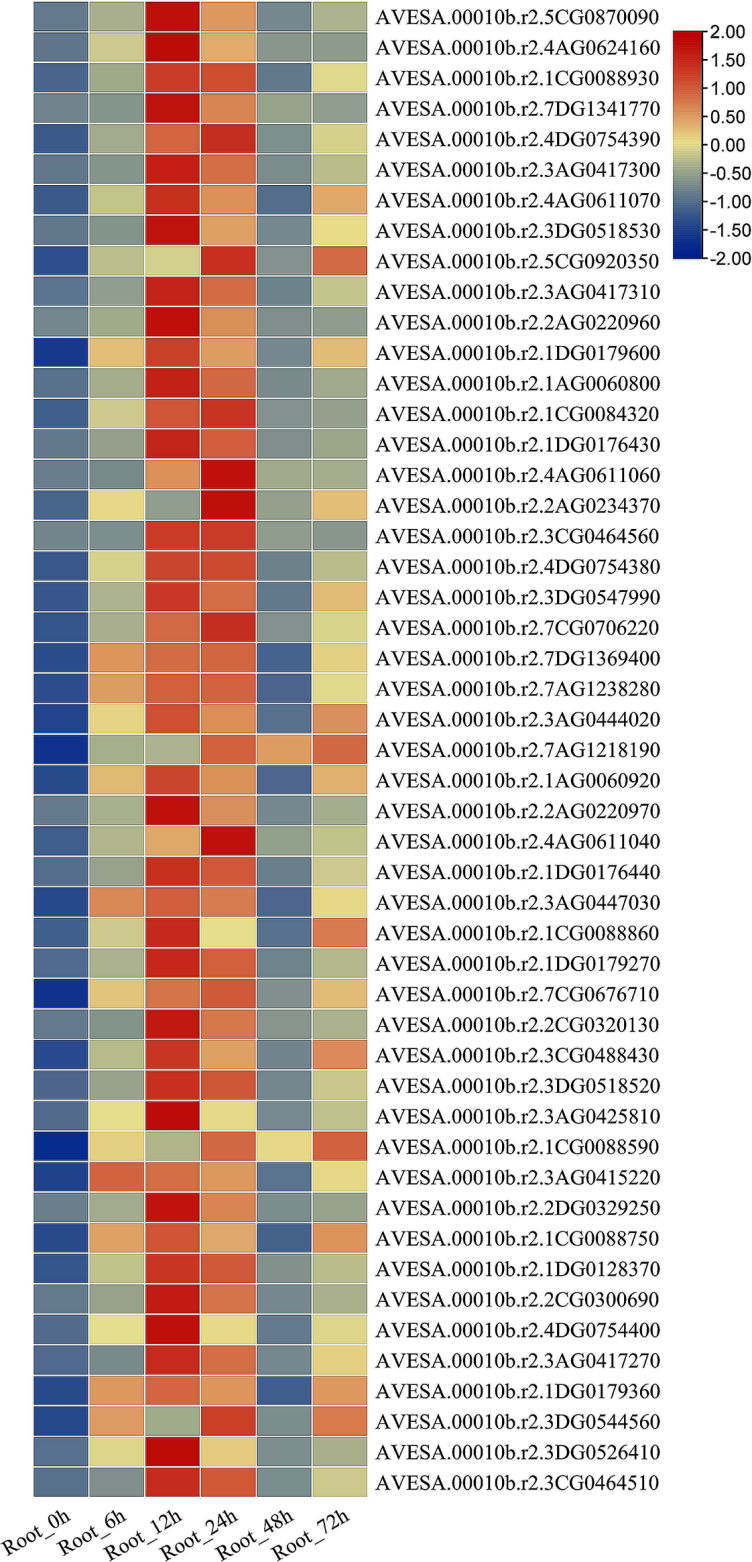
Transcriptional abundance plots of *GST* genes. The heatmap showed the expression profile of *AsMYB1R* TFs. Rows represent the different time samples (0–72 h), the columns represent the different *GST* genes

In transcriptome data, the expression level of the non-enzymatic antagonists *GST* family genes with ROS scavenging function increased after 6 h of PEG treatment, and the expression level of *GST* genes increased starting from 6 h of PEG treatment. Most *GST* genes were upregulated at 12 and 24 h of PEG treatment, but their expression was suppressed at 48 h, only *AVESA.00010b.r2.7AG1218190* gene expression significantly increased. Most genes showed increased expression at 72 h (Fig. 11).

## Discussion

Plants scavenge ROS accumulation due to drought stress through enzymatic and non-enzymatic antioxidant systems, reducing membrane ester peroxidation and avoiding damage to biomolecules such as DNA and proteins [44]. Drought-exposed plants also retain water by increasing amounts of osmolytes to maintain cell turgor pressure and reducing stomatal size to avoid water loss [45]. Reducing stomatal density and increasing the thickness of the wax layer are also essential to reduce water loss via residual (non-stomatal) transpiration [5, 7]. TFs may play an important roles in these processes.

TFs specifically bind to the nucleotide sequence in the promoter region of downstream target genes to activate gene transcription and expression, and then participate in signal transduction and gene expression regulation under abiotic stress. The MYB family is one of the largest TFs families in plants [44]. Studies have shown that *OsMYB2* regulates accumulation of organic osmolytes in rice [46], and *OsMYB48-1* and *BnMYB2-1* were essential for regulation of ABA synthesis in rice [47] and wheat [48], respectively. The *OsMYB60* regulates cuticle wax synthesis in rice [49], and overexpression of *GbMYB5* enhanced the accumulation of proline and antioxidant enzymes in transgenic tobacco, while reducing the production of MDA and improving tobacco drought tolerance [45]. *A. sativa* contain a large number of MYB transcription factors [9, 17], but it is unknown whether the MYB family contribute to drought tolerance. 268 *AsMYB* TFs were identified from the *A. sativa* genome data. Among them, 8 AsMYB TFs differentially expressed under drought stress were identified and analyzed by bioinformatics.

### The *AsMYB2R* subfamily genes in *A. sativa* are related to the shortened growth period, stomatal closure, and nutrient and water transport by PEG-induced drought stress

*AsMYB2R039* is homologous with *AtMYB2R56* in *Arabidopsis*. *AtMYB2R56* is one of the important members of S31 subfamily (Fig. 3), which is confirmed to code carbon starred anthers (CSA). Its role is to promote sugar transport to pollen by binding to the MST8 sugar transporter promoter to control carbon allocation during anther and pollen maturation during rice flowering [50], and it is further confirmed to regulate the size and shape of *Arabidopsis* seeds during seed maturation [51]. Studies have also shown that the interaction between *MYB56* and MATH-BTB/POZ (BPM) protein acts as a negative regulator of flowering in *Arabidopsis* [52]. It is interesting that the expression of *AsMYB2R039* began to increase after PEG treatment, which we believe may be evidence that *A. sativa* prepared to escape drought stress by early initiation of flowering and fruiting mechanisms, and completing the growth cycle ahead of schedule. The results of this study are more likely to be that AtMYB2R56 promotes sugar transport to reproductive organs, promotes early flowering, and shortens the growth cycle in response to drought stress.

The amino acid sequences were analyzed using the Blastp tool on the NCBI website, and the results showed that *AsMYB2R043* and *AtMYB2R61* sequences were highly homologous, with *AtMYB2R61* being a member of the Arabidopsis S16 subfamily (Fig. 3). Effective control of stomatal aperture may be one of the key means for plants to adapt to drought [53]. It was shown that *AtMYB2R61* can reduce stomatal aperture and water loss to resist drought stress [54]. Based on RT-qPCR results, this study found that the expression of *AsMYB2R043* in roots showed a decreasing trend during PEG treatment, while the expression slightly increased at 72 h. However, after drought treatment, the expression of *AsMYB2R043* was inhibited in the leaves (Fig S4). The observation of stomata further confirmed that stomatal aperture continued to decrease with the increase of PEG treatment time, and until 72 h of PEG treatment, the stomata were almost completely closed, thereby reducing excessive water loss. It has been reported that some genes of the *MYB* family are involved in or regulate stomatal closure. Overexpression of *AtMYB61* enhances stomatal closure [55], ABA can induce the expression of *AtMYB44* to promote stomatal closure [56], and overexpression of *AtMYB96* promotes stomatal closure. The stomatal aperture of leaves becomes smaller [57]. *AtMYB60* is involved in regulating stomatal movement, and *AtMYB60* mutants promote stomatal closing [58]. Interestingly though, in the transcriptome data of this study, no above-mentioned homologous genes were differentially expressed (Fig. 10A, Fig S3A and S3B). Therefore, we conclude that the closure of *A. sativa* stomata under drought stress is not regulated by *AsMYB2R043* gene. These conclusions may be one of the important reasons why *A. sativa* can maintain long-term drought resistance.

The *AsMYB2R045* is homologous to *AtMYB2R036* and is a member of the S11 subfamily (Fig. 3). *AtMYB2R036* has been shown to regulate the formation of Casparian strips in the root endothelial layer, which can transport essential nutrients and water for plant growth and development [59]. The *AsMYB2R045* in *A. sativa* roots showed a higher expression level at 0 h, which is consistent with the normal growth and development process of plants that regulate nutrient and water transport through positive regulation of Casparian strips. However, after PEG treatment, the *AsMYB2R045* showed a declining trend in transcriptional levels, indicating that drought inhibited the absorption of nutrients and water in *A. sativa* roots.

### The *AsMYB1R* subfamily genes of *A. sativa* and GST regulation of ROS homeostasis confer signal transduction and scavenge excess ROS to avoid oxidative damage

PEG-induced drought stress upregulated the expression of 5 genes in the *AsMYB1R* subfamily of *A. sativa* (Fig. 9), all of which are key genes in the circadian clock. Among them, *AsMYB1R022*, *AsMYB1R079*, and *AsMYB1R088* are homologous to *REVEILLE 1* (*REV 1*), *AsMYB1R078* is homologous to *REVEILLE 6* (*REV 6*), and *AsMYB1R098* is homologous to *LHY*. *REVEILLE 6* (*REV 6*) which, as an activator of the nocturnal clock, promotes downregulation of the *Arabidopsis* clock gene complex *LHY/CCA1* expression [60]. Upregulation of *AsMYB1R078* (*REV 6*) gene expression may be used to regulate the expression of the *A. sativa* clock complex *LHY/CCA1* genes under drought stress. However, the *MYB1R* subfamily genes are closely related to ROS, studies have shown that the loss of function of the *MYB1R* subfamily genes *CCA1* and *LHY* impairs the production and clearance of ROS in *Arabidopsis* mutants at specific times of the day, suggesting regulatory effects of *CCA1* and *LHY* on basal ROS levels [18], *LHY/CCA1* is one of the main components of the core circular clock machinery complex [61]. The upregulation of *A. sativa REV 1* genes *AsMYB1R022*, *AsMYB1R079*, and *AsMYB1R088* after PEG treatment may be similar in function to the *Arabidopsis MYB1R* subfamily *RVE 1*, acting as clock output genes in *Arabidopsis* and regulating auxin production [62, 63]. It is worth noting that the *RVE 1* gene binds directly to the protochlorophyllide oxidoreductase (PORA) promoter through the EE [(A) AATATCT] - box cis regulatory element. By regulating the catalytic activity of PORA, *RVE 1* promotes the reduction of protochlorophyll (Pchlide) to chlorophyll, avoiding excessive accumulation of Pchlide or a decrease in POR activity during the dark period, thus preventing excessive production of ROS under light stimulation and the ROS-induced oxidative damage [64]. Therefore, the key genes *LHY* (*AsMYB1R098*) and *RVE 1* (*AsMYB1R022*, *AsMYB1R079*, and *AsMYB1R088*) in *A. sativa* circadian clock may be involved in regulation of ROS homeostasis, of which the latter is a key signaling substance regulating plant circadian clock [65]. Changes in ROS content may serve as evidence for *A. sativa* response to drought stress signaling substances.

Through physiological evidence, we further confirmed that the content of H_2_O_2_ in the roots, one of the major types of ROS species, exhibited oscillatory changes in response to PEG treatment (Fig. 10B), which may be used to stabilize the impact of drought stress on the circadian clock system. On the other hand, changes in H_2_O_2_ content are used to provide signal molecules for circadian rhythms and other physiological processes [18, 31, 32, 65]. Furthermore, if H_2_O_2_ content continues to increase, it can oxidize and damage *A. sativa* cell membranes and chloroplasts, induce membrane lipid peroxidation, produce toxic metabolites such as malondialdehyde, damage proteins, lipids, and nucleic acids, and lead to cell death [66]. However, the H_2_O_2_ content in *A. sativa* decreased after 12 and 48 h of PEG treatment (Fig. 10B), which may be partly due to the upregulation of the AsMYB1R subfamily genes *LHY* (*AsMYB1R098*) and *RVE 1* (*AsMYB1R022*, *AsMYB1R079*, and *AsMYB1R088*) on the steady-state regulation of ROS.

We found that genes encoding enzymatic antioxidants such as CAT, SOD, APX did not show differential expression (data not shown). However, the expressions of non-enzymatic antioxidant *GST* genes were significantly upregulated at 12 and 24 h after PEG treatment (Fig. 11), which may be mainly used to scavenge excess ROS produced by sudden outbreaks in the early stages of drought stress. Interestingly, the expression level of *GST* genes showed an up-regulated down-regulated up-regulated oscillation pattern after PEG treatment (Fig. 11), and GST activity in the roots also shows an oscillatory upward pattern (Fig. 10C). These two correlated results are consistent with the long-term changes in the wave pattern of H_2_O_2_ content (in units of h) (Fig. 10B), which not only confirms the continuous regulation of H_2_O_2_ by *A. sativa GST* family genes from transcription to expression, but also matches the signal transduction pattern of ROS waves [32, 67]. It can be, therefore, suggested that *GST* family genes and *MYB1R* subfamily genes jointly regulate H_2_O_2_ levels. An appropriate amount of H_2_O_2_ is used for signal transmission in plants, while excessive H_2_O_2_ can cause oxidative damage to plants. Therefore, we infer that the *GST* gene family plays a major role in regulating H_2_O_2_ content. The *MYB1R* subfamily genes *CCA1*, *LHY* and *REV1* are important clock genes, and there is evidence that a loss of *CCA1* and *LHY* function impairs ROS production and scavenging in Arabidopsis mutants at specific times of the day, and that the *RVE1* gene can bind to the promoter of protochlorophyllide oxidoreductase (PORA) to regulate ROS levels [64]. Therefore, we infer that the respectively up-regulated expression of *LHY* (*AsMYB1R098*) and *RVE 1* (*AsMYB1R022*, *AsMYB1R079* and *AsMYB1R088*) of A. sativa may be involved in the regulation of ROS homeostasis.

## Conclusions

In this study, a genome-wide identification of *MYB* genes in *A. sativa* was performed and a total of 268 *AsMYB* genes were identified. The expression levels of eight TFs in *A. sativa* roots by PEG-induced drought stress at different time points were analyzed based on transcriptome data and RT-qPCR. Of these, three genes from *AsMYB2R* subfamily (*AsMYB2R039*, *AsMYB2R043*, *AsMYB2R045*) played essential role in the shortening growth period, triggering stomatal closure, and controlling nutrient and water transport under PEG drought stress. The results confirmed that the upregulation of key *AsMYB1R* subfamily genes *LHY* (*AsMYB1R098*) and *RVE 1* (*AsMYB1R022*, *AsMYB1R079*, and *AsMYB1R088*) in *A. sativa* under PEG stress may be used to regulate ROS homeostasis, and ROS may be a key signaling substance for the circadian clock. GST, as a non-enzymatic antioxidant, works together with the key genes *LHY* (*AsMYB1R098*) and *RVE 1* (*AsMYB1R022*, *AsMYB1R079*, and *AsMYB1R088*) in *A. sativa* to regulate ROS homeostasis and maintain the basal H2O2 level required for signal transduction while avoiding oxidative damage to cells.

## Materials and methods

### Screening of the AsMYB TFs and physicochemical properties analysis

The genome and annotation data of *A. sativa* were downloaded from the GrainGenes database (https://wheat.pw.usda.gov/GG3/content/avena-sang-download). Characteristic Hidden Markov Model (HMM) and alignment files of the MYB protein structural domain (PF00249) from the PFAM database (https://pfam-legacy.xfam.org) were used as search files to initially identify the candidate *AsMYB* genes (E-values < 1 × 10^− 5^) by HMMER software (Version 3.0). Then, to remove redundant sequences, *AsMYB* genes were further screened by using CD-HIT software (Version 4.8.1) with the parameters c = 0.9, *n* = 5. In some cases, multiple transcripts of *AsMYB* genes were noticed, and only the longest transcript corresponding to each *AsMYB* gene was retained for further studies [68]. Finally, the candidate *AsMYB* genes were further manually examined for the completeness of the conserved domain in the protein sequence using the NCBI conserved domain database (https://www.ncbi.nlm.nih.gov/cdd/) and search against the simple modular architecture research tool (SMART) website (https://smart.embl.de) with default parameters (E-values < 1 × 10^− 5^). Only genes with intact MYB conserved domain can be used for subsequent analysis. Based on the number and the characteristic of *AsMYB* genes imperfect tandem repeat, *AsMYB* genes were classified into different categories. Alignment file of amino acid sequences aligned by Clustal X software (Version 2.1) were submitted to Weblogo online website (https://weblogo.berkeley.edu/logo.cgi) to exhibit the *MYB* imperfect tandem repeat sequences. The physicochemical properties of AsMYB proteins, including protein size, molecular weight, isoelectric point, instability index, and grand average of hydropathicity, were analyzed by using the online program ExPASy-ProtParam (https://www.expasy.org/resources/protparam). Furthermore, the subcellular localization of the *AsMYB* genes was predicted using the online tool WOLF PSORT (https://wolfpsort.hgc.jp).

### Chromosome localization and phylogenetic tree construction

The location of *AsMYB* genes in different categories on the chromosomes were obtained from the *A. sativa* annotation data using the Gene Location Visualize program of TBtools software (Version 2.003) [69]. To facilitate the subsequent research, the location of *AsMYB* genes were ensured and systematically named according to the order on the chromosome. Subsequently, to examine the phylogenetic relationship and evolutionary history of *AsMYB* genes, the identified AsMYB proteins were combined with the reported MYB proteins of *A. thaliana* [70], which were obtained from TAIR database (http://www.arabidopsis.org/) to construct protein alignment files by using Clustal X software. Subsequently, the Neighbor-Joining phylogenetic tree was built from the alignment files using MEGA software (Version 11.0) with the following parameters: p-distance, partial deletion (50%), and bootstrap analysis with 1000 replicates, respectively. Finally, the phylogenetic tree was modified using the Interaction Tree of Life (iTOL) online website (https://itol.embl.de).

### Conserved motif, gene structure, and cis-acting analysis

To further investigate conserved motifs of AsMYB proteins, the conserved motifs of amino acid sequences were analyzed by using the MEME online program (https://meme-suite.org/meme/tools/meme). The number of conserved motifs searched was 10, and the rest of the settings were left unchanged. For a visual visualization, the evolutionary tree file, conserved motifs, genome annotation data, and the positions of MYB binding site which extracted by CD-search tool (https://www.ncbi.nlm.nih.gov/cdd/) were submitted to Gene Structure View program of TBtools software.

To analyze the cis-acting elements in the promoter region, the 2000 bp length of the upstream DNA sequences of *AsMYB* TFs were submitted to the PlantCARE database (https://bioinformatics.psb.ugent.be/webtools/plantcare/html/).

### Gene duplication and collinearity analysis

Gene duplication events of *AsMYB* genes were detected using One Step MCScanX program (E-values < 1 × 10^–10^) of TBtools software with default parameters and visualized the location of *AsMYB* genes, gene density, and collinearity information of *AsMYB* genes, the Advance Circos program was chosen to perform the work. The selection pressure in biological evolution can be represented by the rate of nonsynonymous and synonymous (Ka/Ks) [71]. Relevant files were entered into Simple Ka/Ks Calculator program of TBtools software to obtain the calculation results.

### Plant materials and treatment

A crop seed detector (BIO seed M-P, Research Center of Information Technology, Beijing Academy of Agriculture and Forestry Sciences, Beijing, China) was used to screen *A. sativa* seeds with consistent phenotypes. Seeds were surface sterilized with sodium hypochlorite (1.5%) for 20 min, then placed in a germination box and cultured in a growth chamber at 25 ± 1℃ until the seedling height was 2–3 cm. Plants were then transferred to a 1/2 Hoagland nutrient solution for further growth [72]. Once the seedlings had two true leaves, nutrient solutions that contain 15% (w/v) polyethylene glycol 6000 (PEG 6000) were supplied [68]. This concentration of PEG has osmotic potential of about 1.2 MPa and was usually used in numerous studies to mimic drought stress. After the certain treated time points (0/6/12/24/48/72 h), the roots were rinsed with sterile water and the residual water droplets were removed, followed by flash freezing with liquid nitrogen. Finally, the roots were stored at -80℃ for further analysis.

### Templet preparation and RT-qPCR

Total RNA was extracted from all roots of *A. sativa* using the RNAiso Plus Kit (Takara, Japan) as the manufacturer’s guidelines. The concentration and purity of isolated total RNA were estimated by Nanodrop spectrometer (Thermo Fisher Scientific, USA). First-strand complementary DNA (cDNA) which using 1 µg of total RNA as the template was synthesized using the PrimeScript^™^ RT reagent kit with gDNA Eraser (Takara, Japan). Gene specific primers for RT-qPCR were designed by Primer Premier software (Version 5.0) and shown in Table S1. RT-qPCR was performed with CFX Connect (Bio-Rad, USA) using ArtiCan^CEO^ SYBR qPCR Mix (Tsingke, China). The reaction system (20 µL) was performed as follows: 10 µL ArtiCan^CEO^ SYBR qPCR Mix, 0.4 µL of each primer (10 µM), 2 µL cDNA (Ten-fold dilution was performed with water), the rest of the volume were replenished by water. The thermal cycle program was set as follows: initial denaturation at 95℃ for 5 min, followed by 40 cycles of 95℃ for 10 s and 60℃ for 30 s, the melt curve of built-in program was set to verify the specificity of the primers. The primer of *GAPDH* gene was chosen as the internal reference gene to normalize cDNA concentrations [73]. The relative expression levels of *AsMYB* genes was calculated by utilizing 2^-ΔΔCq^ algorithm.

### Physiological traits

The *A. sativa* leaves were excised from 6 time point samples, and an EVOS microscope (Thermo Fisher Scientific, USA) was used to observe the plant stomatal status in the abaxial surface of the blade. In Fig S3A, the stomatal width of differential photos was measured by ImageJ program [74]. The content in the roots of H_2_O_2_ was determined using Hydrogen Peroxide (H_2_O_2_) Content Assay Kit (Solarbio, China) following the manufacturer’s protocol. The roots (0.1 g) were homogenized in 1 mL of cold acetone, multiple regents were added to mixture and its absorbance was measured at 415 nm. The TissueLyser II machine (QIAGEN, Germany) was used for tissue fragmentation [75]. GST activity in roots was determined following the protocol of the Glutathione S-transferase (GST) Activity Assay Kit (Solarbio, China) [76]. The roots (0.1 g) were homogenized on ice with 1 mL regent 1 of GST Activity Assay Kit. The absorbance was measured at 340 nm after multiple reagent treatments.

### Statistical analysis

Three independent sample replicates were used for RT-qPCR analysis at each time point, and six independent sample replicates were used for H_2_O_2_ content and GST activity analysis at each time point. All the statistical analyses were performed using GraphPad Prism9 (GraphPad Software Inc.; San Diego, CA, USA). The reported data are presented as mean ± SD (Standard Deviation). One-way ANOVA is used to analyze the significance of different treatments (*p* < 0.05 significance level).

### Electronic supplementary material

Below is the link to the electronic supplementary material.

Supplementary Material 1

Supplementary Material 2

## Data Availability

All RNA-Seq data were deposited in the NCBI SRA database under the project PRJNA1056521 (https://www.ncbi.nlm.nih.gov/bioproject/PRJNA1056521).
