# Supplementary material for: Genome-wide analysis of MYB transcription factor family and AsMYB1R subfamily contribution to ROS homeostasis regulation in Avena sativa under PEG-induced drought stress
Source: BMC Plant Biol. 2024 Jul 6;24:632. doi: 10.1186/s12870-024-05251-w (PMC11227197; doi:10.1186/s12870-024-05251-w)
Supplement: Supplementary file 2 — Supplementary Material 2 [file 12870_2024_5251_MOESM2_ESM.pdf]

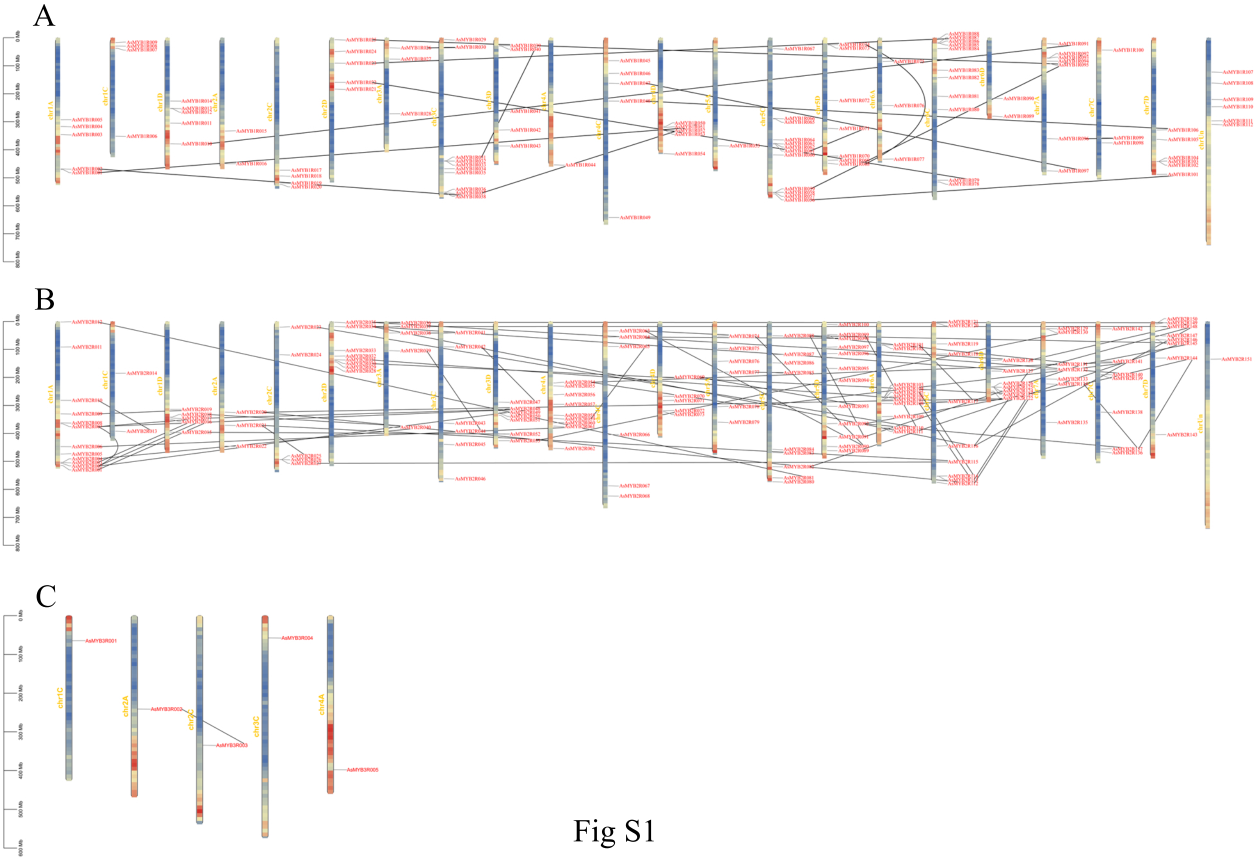

Fig S1

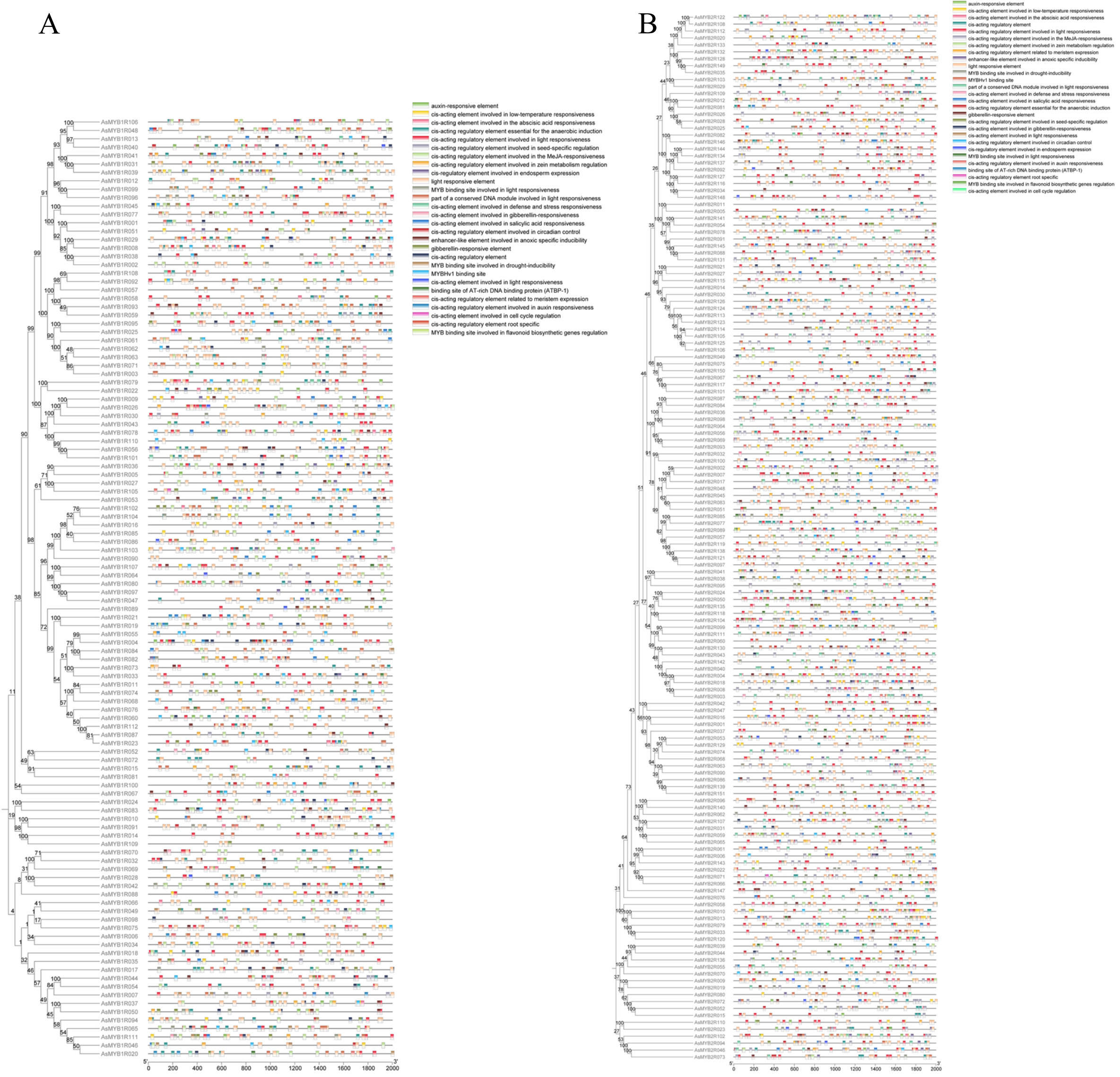
A
B
auxin-responsive element
cis-acting element involved in low-temperature responsiveness
cis-acting element involved in the abscisic acid responsiveness
cis-acting regulatory element essential for the anaerobic induction
cis-acting regulatory element involved in light responsiveness
cis-acting regulatory element involved in seed-specific regulation
cis-acting regulatory element involved in the MeJA-responsiveness
cis-acting regulatory element involved in zein metabolism regulation
cis-regulatory element involved in endosperm expression
light responsive element
MYB binding site involved in light responsiveness
part of a conserved DNA module involved in light responsiveness
cis-acting element involved in defense and stress responsiveness
cis-acting element involved in gibberellin-responsiveness
cis-acting element involved in salicylic acid responsiveness
cis-acting regulatory element involved in circadian control
enhancer-like element involved in anoxic specific inducibility
gibberellin-responsive element
cis-acting regulatory element
MYB binding site involved in drought-inducibility
MYBHv1 binding site
cis-acting element involved in light responsiveness
binding site of AT-rich DNA binding protein (ATBP-1)
cis-acting regulatory element related to meristem expression
cis-acting regulatory element involved in auxin responsiveness
cis-acting element involved in cell cycle regulation
cis-acting regulatory element root specific
MYB binding site involved in flavonoid biosynthetic genes regulation
5'
3'
0
200
400
600
800
1000
1200
1400
1600
1800
2000

Fig S2

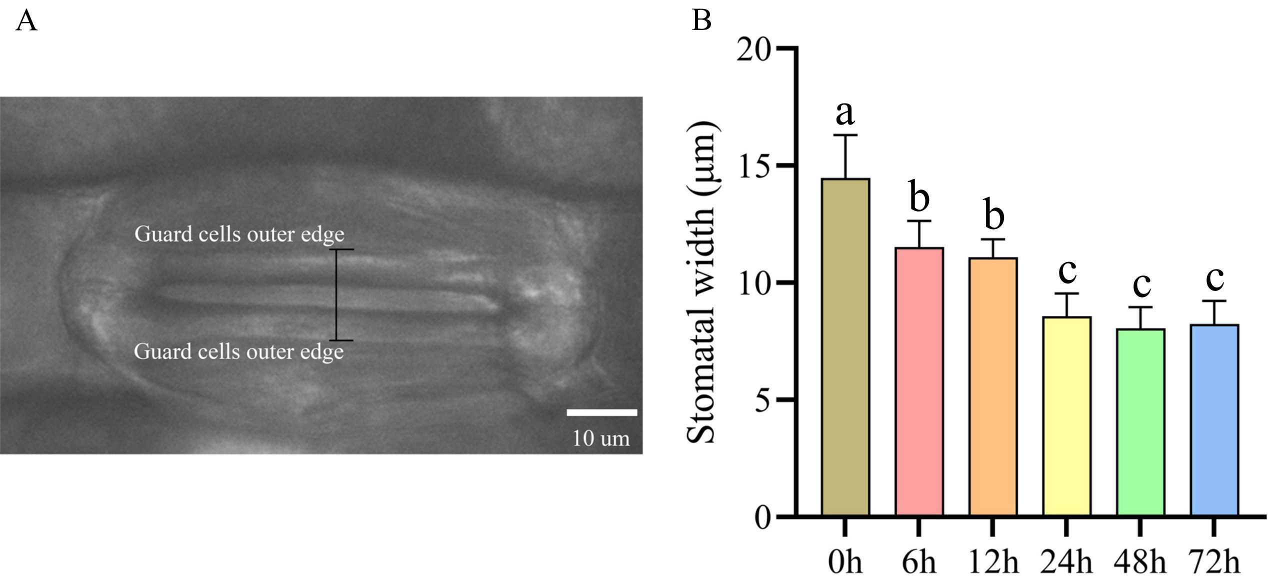
A
Guard cells outer edge
Guard cells outer edge
10 um
B
Stomatal width (μm)
20
15
10
5
0
a
b
b
c
c
c
0h
6h
12h
24h
48h
72h

Fig S3

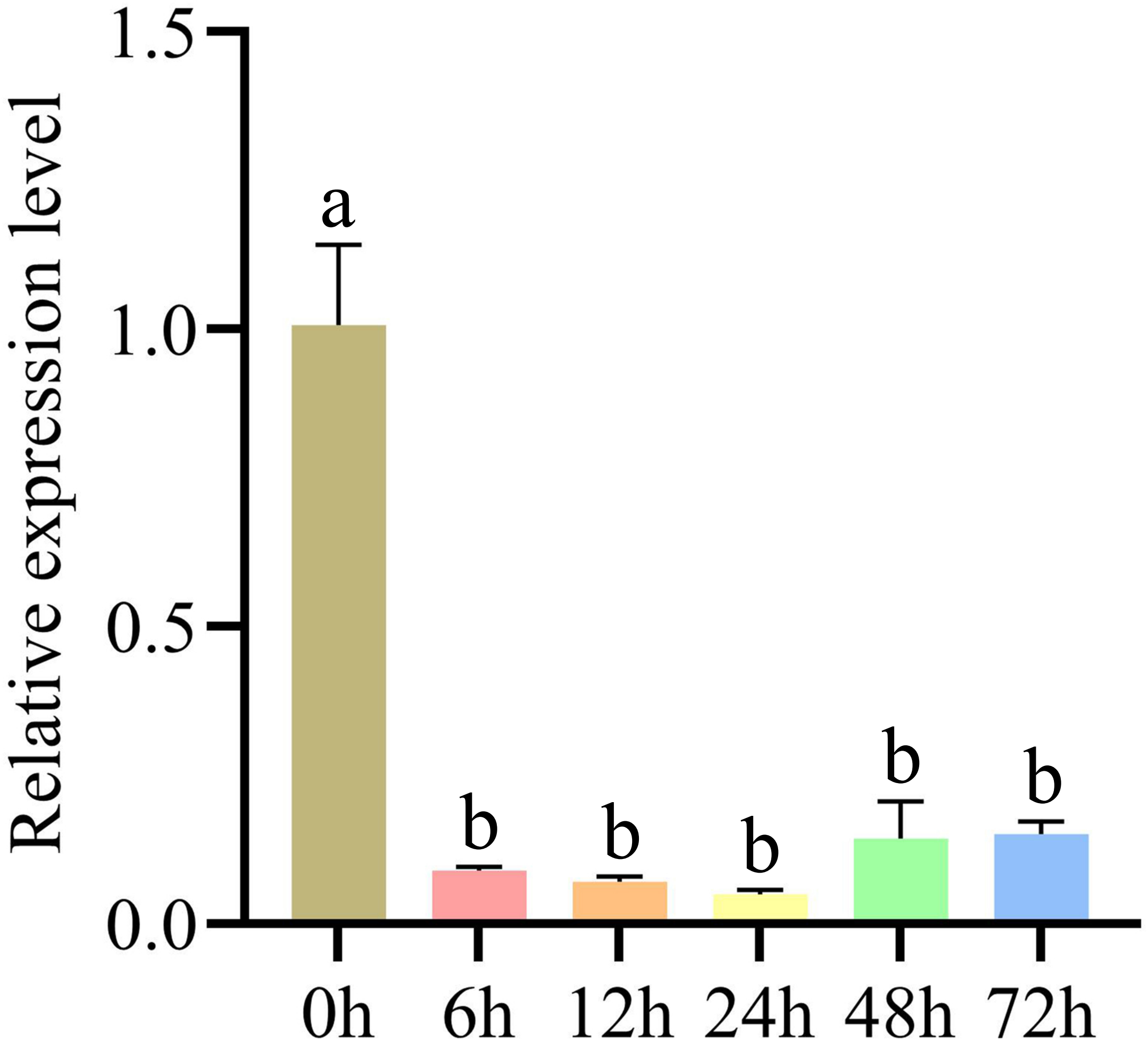

Relative expression level
1.5
1.0
0.5
0.0
a
b
b
b
b
b
0h
6h
12h
24h
48h
72h

Fig S4

Fig. S1. The duplication information of *AsMYB* TFs on *A. sativa* chromosomes. (**A**) The information of *AsMYB1R* TFs. (**B**) The information of *AsMYB2R* TFs. (**C**) The information of *AsMYB3R* TFs.

Fig. S2. Distribution of cis-acting elements in the promoter regions of the *AsMYB* TFs. (**A**) The cis-acting elements of *AsMYB1R* TFs. (**B**) The cis-acting elements of *AsMYB2R* TFs.

Fig. S3. The example diagram of stomatal width calculation and the change of stomatal width at 6 time points (0-72 h). (**A**) The example diagram of stomatal width calculation; The distance between two stomatal guard cells outer edge represents stomatal width and the size of scale bar is 10 μm. (**B**) stomatal width at 6 time points (0-72 h). Mean ± SD (Standard Deviation) was obtained from six biological replicates. The error bars indicate standard deviation. Different letters indicate significant differences, and the same letters represent no significant differences at the 0.05 level.

Fig. S4. The RT-qPCR plots of *AsMYB2R043* gene. Relative expression level plots of *AsMYB2R043* gene in PEG treated leaves was determined by RT-qPCR. The Y-axis and X-axis indicated relative expression level and six time points of PEG treatment in leaves, respectively. The relative expression level of genes at 0 h was taken as 1, and were calculated by normalization method. Mean ± SD (Standard Deviation) was obtained from three biological and three technical replicates. The error bars indicate standard deviation. Different letters indicate significant differences, and the same letters represent no significant differences at the 0.05 level.
